# The Role of Decorin and Biglycan Signaling in Tumorigenesis

**DOI:** 10.3389/fonc.2021.801801

**Published:** 2021-11-30

**Authors:** Valentina Diehl, Lisa Sophie Huber, Jonel Trebicka, Malgorzata Wygrecka, Renato V. Iozzo, Liliana Schaefer

**Affiliations:** ^1^ Institute of Pharmacology and Toxicology, Goethe University, Frankfurt, Germany; ^2^ Department of Internal Medicine I, Goethe University, Frankfurt, Germany; ^3^ Center for Infection and Genomics of the Lung, Member of the German Center for Lung Research, University of Giessen and Marburg Lung Center, Giessen, Germany; ^4^ Department of Pathology, Anatomy and Cell Biology and the Translational Cellular Oncology Program, Sidney Kimmel Cancer Center, Sidney Kimmel Medical College at Thomas Jefferson University, Philadelphia, PA, United States

**Keywords:** extracellular matrix, proteoglycan, autophagy, inflammation, angiogenesis, cancer, toll-like receptor

## Abstract

The complex and adaptive nature of malignant neoplasm constitute a major challenge for the development of effective anti-oncogenic therapies. Emerging evidence has uncovered the pivotal functions exerted by the small leucine-rich proteoglycans, decorin and biglycan, in affecting tumor growth and progression. In their soluble forms, decorin and biglycan act as powerful signaling molecules. By receptor-mediated signal transduction, both proteoglycans modulate key processes vital for tumor initiation and progression, such as autophagy, inflammation, cell-cycle, apoptosis, and angiogenesis. Despite of their structural homology, these two proteoglycans interact with distinct cell surface receptors and thus modulate distinct signaling pathways that ultimately affect cancer development. In this review, we summarize growing evidence for the complex roles of decorin and biglycan signaling in tumor biology and address potential novel therapeutic implications.

## 1 Introduction

### 1.1 Extracellular Matrix Guidance in Tumor Initiation and Progression

Over the past decades, it has become apparent that tumorigenesis is not merely the result of accumulated DNA mutations, but tumor development and progression also depend on the context in which malignant cells subsist (1–3). The tumor microenvironment (TME) is a permissive infrastructure primarily composed of non-malignant resident cells, cancer-associated fibroblasts, macrophages, and a variety of inflammatory and immune cells (4–7), pericytes (8, 9), and cells of the tumor-associated vascular system (10, 11). Moreover, the TME includes the extracellular matrix (ECM), an acellular structure with, as has become evident by now, unique tumor-specific characteristics (12, 13).

The ECM forms an intricate, highly ordered, 3D meshwork of large macromolecules, the components of which have been considered in the past solely as structural scaffolds that enable cellular adhesion and migration. However, a vast body of emerging evidence has led to a paradigmatic shift implicating ECM proteins in various signaling pathways and cellular processes including autophagy, inflammation, proliferation, survival, cell morphology, and motility (14–22). To date, several ECM components are increasingly appreciated for mediating and modulating cellular signaling (18, 23–26), and presumably even inter-organ cross-talk (27). Importantly, changes in the ECM are known to lead to, or to correlate with several diseases such as cardiovascular and skeletal disorders, fibrosis and importantly cancer (24, 28–30). The observations that link the ECM to cancer are as diverse as the different macromolecules that comprise the ECM. The impact of the biochemical composition of the ECM on tumor progression (12, 31–33), the ECM-dependent development of metastatic niches (34, 35), and the signaling between the tumor-adjacent ECM and the different players of the TME have been reviewed in detail (36, 37). In this review, we will focus on two proteoglycans of the ECM, biglycan and decorin. We will discuss their implications in selected pathways that are linked to the hallmarks of cancer, with special emphasis on autophagy and inflammation, whose contribution to tumorigenesis is increasingly recognized. Lastly, we will critically discuss recent advances and future perspectives in therapeutic targeting of these proteoglycans for cancer therapy.

### 1.2 Inflammation as an Emerging Hallmark of Cancer

Tumorigenesis is a multi-factorial process that is characterized by specific biological capabilities that are acquired during the development of tumors. These hallmarks of cancer include evading apoptosis, increased angiogenesis, insensitivity to anti-growth signals, and tissue invasion and metastasis (38). In addition to these established hallmarks of cancer, the vital roles of inflammation and the immune response have gained recognition as emerging hallmarks and enabling characteristics of cancer (31, 39, 40). Intricate interactions between cells of the tumor proper, surrounding stromal cells, and inflammatory cells result in an inflammatory TME. Indeed, chronic tissue inflammation has been linked to a heightened risk for malignant transformation, and additionally, sustained inflammation is assumed to cause pre-cancerous lesions (41–43). Moreover, it is well-established that the TME of most tumors is characterized by the presence of highly abundant inflammatory cells and inflammatory mediators, irrespective of the stages of tumor progression (44). Generally, there are two different types of cancer-associated inflammation. One can exert tissue-protective, anti-tumoral functions; the second on the other hand, is linked to pro-tumorigenic effects, presumably in a context and spatiotemporal-dependent manner (45).

Historically, cancer-associated inflammation was attributed to an attempt by the host immune system to eliminate the tumor and provide tissue-protection. Indeed, for some tumor types, there is increasing evidence for an acute immune response against the tumor, and the primary tumor development depends on the ability of tumor cells to escape the immune-mediated destruction (46, 47). This concept has been exploited for the development and application of immuno-therapies that reinstall or enhance the patient’s immune response towards recognizing and eradicating cancer cells (48, 49). Conversely, the tumor-associated inflammatory response can also facilitate tumorigenesis by providing an inflammatory milieu. This “cancer-promoting inflammation” relies on secretion of growth factors and enzymes to sustain proliferation, inhibit cell death, promote epithelial-mesenchymal transition (EMT), facilitate angiogenesis, invasion, and metastasis (39, 45, 50). In line with this, chronic inflammation has been linked to cancer-promoting effects in so-called ‘hot’ tumors, which are densely infiltrated by immune cells and are often unresponsive to immuno-therapy (44, 45, 51).

On a molecular level, constitutively active NF-κB (nuclear factor kappa-light-chain-enhancer of activated B-cells) signaling is considered to be one of many possible factors that promote chronic inflammation in the context of tumorigenesis (52). NF-κB downstream signaling results in expression of inflammatory cytokines, adhesion molecules, the inducible nitric oxide synthase and angiogenic factors. Inducers of NF-κB include cytokines such as tumor necrosis factor alpha (TNF-α) and interleukin 1 beta (IL-1β). The activation of the NF-κB pathway is mediated through Toll-like receptors (TLRs) that act in concert with their adaptor molecules and co-receptors (53–55). Another signaling pathway that links inflammatory signaling to tumorigenesis includes the NOD-like receptor protein 3 (NLRP3) inflammasome, which activates NOD-like receptors or AIM2-like receptors and ultimately promotes the release of the inflammatory cytokines *via* activating Caspase 1, however, its precise function in tumor biology remains controversial (56–58).

In summary, inflammation and immune responses can critically contribute to the cancer onset and determine the outcome of the disease progression. Therefore, inflammation can be considered an enabling characteristic of cancer (39). Investigating the role of proteoglycans in mediating these cancer-associated inflammatory processes may provide novel insights in tumor biology.

### 1.3 Emerging Implications of Autophagy in Tumorigenesis

Autophagy has an ambivalent role in cancer development and progression. Depending on several factors, such as the tumor progression state, the TME and the genetic context, it can exert opposite outcomes and promote or decrease tumorigenesis (59–61). On one hand, before the onset of malignant transformation and tumorigenesis, the cytoprotective effect of autophagy can promote tumor suppression (62–65). On the other hand, evidence suggest autophagy to promote the survival of cancer cells under certain stress conditions, including therapy-induced stress. Moreover, various cancer types were shown to be linked to a high autophagy (66), such as cancers associated with *RAS* (Ras GTPase, Rat sarcoma) mutations (67, 68), or pancreatic cancer (66, 69), which have been reported to exhibit a high autophagic dependency. Additionally, the upregulation of autophagy is considered to be a frequent side effect of cancer therapies. Therefore, diverse autophagy inhibitors were suggested to increase the efficiency of therapeutics by reducing the tumor cells autophagy-dependent adaptive response mechanisms (70–72). However, increased therapy efficiencies have has also been reported upon activation of autophagy (73). Furthermore, unconventional secretion, endosomal and exosomal pathways have been shown to link autophagy to affecting cancer metastasis, immune responses and shaping the TME (74). Additionally, cross-talk between autophagy and the EMT was reported (75). In this context, autophagy induction was shown to impair EMT (76, 77), which is a critical contributor to metastasis. Other studies, however, showed that treatment-induced autophagy could exert cyto-protective functions and promote melanoma cell EMT (78). In line with this, knockdown of Beclin-1, which is required for autophagosome formation, reduced EMT of colon cancer cells (79). Notably, autophagy activation was also associated with increased anoikis resistance and enhanced metastasis in several tumor models (80, 81).

Several autophagy inhibitors and inducers are currently being evaluated as treatment options in cancer therapies (82, 83), however, the ambiguous roles of autophagy in tumor biology and the highly context-dependent outcomes of modulating autophagy in different cancer types and progression stages impede the translation to clinical applications. A deeper understanding of how proteoglycans influence autophagy in cancer might allow more precise indications for autophagy inhibitors and inducers, and enable the development of adjuvant therapies.

### 1.4 Structural Characteristics of Small Leucine-Rich Proteoglycans

Proteoglycans are complex molecules that are embedded in the ECM and comprise a protein core and one or more glycosaminoglycan (GAG) chains, which are covalently tethered to the protein core. The different types of GAG chains can be formed by heparan sulfate, chondroitin sulfate/dermatan sulfate (CS/DS), or keratan sulfate (84–86). In mammalian cells, more than 40 proteoglycans have been described, which are classified according to their localization into extra-, peri-, and intracellular, as well as plasma membrane-associated proteoglycans (85). The divers and manifold biological roles of proteoglycans can be attributed to their variability and modularity, as their protein cores and the heterogeneous GAG chains can be variously modified. Proteoglycans critically provide structure to the ECM, are key receptors to diverse signaling stimuli, such as growth factors, cytokines, chemokines, and important regulators at the intersection of the cell and matrix (87, 88).

The complex and multifaceted regulatory functions of proteoglycans are well exemplified by their largest protein family, the small leucine-rich proteoglycans (SLRPs), which are ubiquitously expressed and highly abundant in the ECM (89). After being secreted into the pericellular space SLRPs are incorporated into the ECM. They are composed of a relatively small protein core of about 40–60 kDa that contains 10–12 motifs of leucine-rich repeats (LRRs) and is flanked by characteristic cysteine-rich clusters with defined spacing. Additionally, the heterogeneous GAG chains are post-translationally attached to the protein core (85, 90–92). To date, there are 18 known SLRPs, which are classified into five distinct classes, based on their N-terminal cysteine-rich clusters, evolutionary conservation, sequence homology, and GAG chains (85). The LRRs of the protein core, which give rise to the characteristic solenoid structure of SLRPs, contain a conserved hydrophobic motif wherein distinct leucines can be substituted by other hydrophobic amino acids (90). Irrespective of their classification, all SLRPs share common biological functions, and most importantly, their capability to interact with various cell surface receptors such as receptor tyrosine kinases (RTKs) and TLRs, thus mediating divers downstream signaling events that regulate vital cellular pathways and processes that are also implicated in cancer, including inflammation and autophagy (17). SLRPs can be released upon tissue stress or injury and can act as damage-associated molecular patterns (DAMPs) (93, 94).

Importantly, an emerging body of evidence points to the vital implications of the SLRPs decorin and biglycan in several hallmarks of cancer and regulating tumor-associated pathways, such apoptosis, proliferation, angiogenesis, inflammation, and autophagy (17, 18, 87, 93–95). In the following sections, we will summarize and discuss the major signaling functions of biglycan and decorin in normal physiology and in the context of cancer.

## 2 Decorin Function In Tumorigenesis

**Figure 1:**
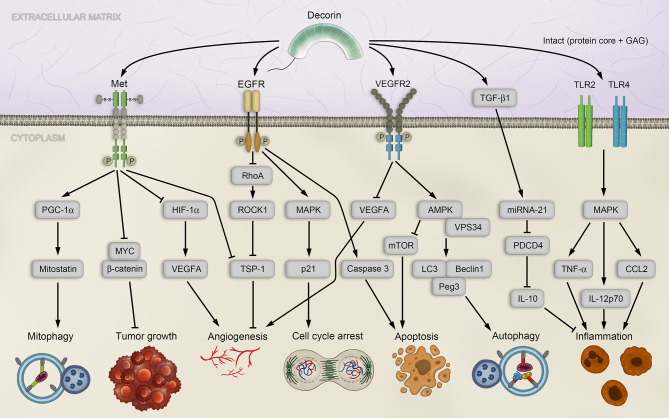
Decorin-mediated signaling affects diverse pathways associated with tumorigenesis. Decorin activates mitophagy in breast carcinoma cells by binding to the Met receptor and activating PGC-1α, leading to accumulation of Mitostatin. Further, *via* Met receptor signaling, decorin inhibits tumor growth by down-regulating β-catenin and MYC, and inhibiting angiogenesis by repressing HIF-1α and VEGFA. Angiogenesis is also modulated by decorin through EGFR in a signaling cascade that employs Rho and ROCK1, leading to upregulation of the anti-angiogenic effector TSP-1. EGFR phosphorylation by decorin induces intracellular MAPK, resulting in enhanced expression of p21 and cell cycle arrest, and in release of Caspase 3 and apoptosis. Decorin-mediated VEGFR2 signaling inhibits angiogenesis and induces autophagy in endothelial cells by inhibiting mTOR, activating VPS34, Beclin-1, LC3 and Peg3. Decorin regulates inflammation in a PDCD4-dependent manner through miRNA-21, or by TLR2/4 signaling. AMPK, AMP-activated protein kinase; CCL, chemokine C–C motif-ligand; EGFR, epidermal growth factor receptor; GAG, glycosaminoglycan; HIF-1α, hypoxia-inducible factor 1α; IL, interleukin; LC3, microtubule-associated protein 1A/1B-light chain 3; MAPK, mitogen-activated protein kinase; Met, mesenchymal-epithelial transition factor; miRNA, microRNA; mTOR, mammalian target of rapamycin; MYC, myelocytomatosis oncogene protein; PDCD4, programmed cell death protein 4; Peg3, paternally expressed 3; PGC-1α, peroxisome proliferator-activated receptor-γ co-activator 1-α; Rho, RAS homolog family member A; ROCK1, Rho-associated coiled-coil kinase 1; TGF-β1, transforming growth factor β isoform 1; TLR, Toll-like receptor; TNF-α, tumor necrosis factor alpha; TSP-1, thrombospondin-1; VEGFA, vascular endothelial growth factor A; VEGFR2, vascular endothelial growth factor receptor 2; VPS34, vacuolar protein sorting 34.

Decorin is a well-characterized, prototypical member of the SLRP family and ubiquitously expressed in most tissues (96). It is mainly known for its vital functions in inflammation, innate immunity, wound healing, fibrotic diseases, angiogenesis, autophagy and cancer, where it is found in the stroma of several cancer types (97–100). Decorin, whose eponym derives from its high affinity for collagen fibrils and thus able to “decorate” collagen fibrils (92, 101, 102), was the first proteoglycan that was associated with regulating the cell cycle by inhibiting transforming growth factor β (TGF-β) signaling (103). This has led to a paradigmatic shift in matrix biology research, since proteoglycans have previously been described only as structural components. Indeed, decorin deficiency in mice leads to a permissive environment favoring tumorigenesis and EMT (104, 105). The diverse functions can be attributed to its ability to interact with numerous components of the ECM and cellular receptors, thereby inducing intracellular signaling cascades. Decorin has the ability to acts as a Pan-RTK inhibitor by engaging numerous RTKs, including the epidermal growth factor receptor (EGFR), the vascular endothelial growth factor receptor 2 (VEGFR2) and the mesenchymal-epithelial transition factor (Met) receptor (**Figure 1**), and can induce caveosomal internalization and degradation of the RTKs, thus restraining angiogenesis. Therefore, and because of its capability to inhibit cancer growth by sequestrating TGF-β, decorin has also been described as “the guardian from the matrix” (99).

### 2.1 Decorin Acts as a Versatile Tumor Suppressor

One of the most significant RTKs, whose downstream signaling is affected by decorin, is the Met receptor pathway (106–108). Met receptor-mediated signaling is vital for embryonic development and upregulation of the receptor has been linked to tumorigenesis and metastasis (109–112). Direct binding of decorin to the Met receptor results in tumor suppression by phosphorylation of Met tyrosine sites (106), leading to recruitment of the proto-oncogene CBL (Casitas B-lineage Lymphoma) and subsequent proteosomal degradation of the Met receptor (113). This prevents binding of the natural ligand of the Met receptor, HGF (hepatocyte growth factor), which can contribute to tumor survival, growth, angiogenesis, and metastasis under pathologic conditions (112). The antagonistic effect of decorin on Met signaling results in down-regulation of the major oncogene β-catenin and its downstream effector MYC (myelocytomatosis oncogene protein), both involved in the pathologies of various cancers (107, 114), and thus promotes an anti-tumorigenic activity of decorin (**Figure 1**).

Apart from engaging the Met receptor, decorin can attenuate tumor growth as a monomeric proteoglycan (115). To date, the protein interacting network centered on decorin is vast and expanding (116), with numerous growth factors, receptors, and various matrix molecules. The interaction of decorin with TGF-β causes strong inhibition of proliferation in various cancer cell lines, presumably by decorin-binding activity on TGF-β isoform 1 (TGF-β1), thereby sequestrating it in the ECM and limiting its bioactivity (117). Mechanistically, EGFR phosphorylation by decorin induces intracellular MAPK (mitogen-activated protein kinase) signaling that results in enhanced expression of the cyclin-dependent kinase inhibitor p21, triggering cell cycle arrest, and in release of Caspase 3 to promote apoptosis (99, 108, 118, 119) (**Figure 1**). Moreover, decorin competes with the natural ligand of EGFR, the epidermal growth factor (EGF), and the decorin-EGFR interaction results in internalization and degradation of the receptor *via* caveolar endocytosis (120), thus controlling tumor growth (121–123).

### 2.2 Decorin Regulates Angiogenesis *via* Receptor Tyrosine Kinase Signaling

A central issue of tumorigenesis is angiogenesis, an intrinsic process required for supplying oxygen and nutrients to the growing tumor through vessels newly formed from preexisting large vessels. The role of decorin in regulating angiogenesis is ambivalent, as decorin promotes angiogenesis by facilitating endothelial cell adhesion and migration in a non-malignant state (108, 124, 125). Likewise, it was shown in healthy, non-tumorigenic models that decorin protects endothelial cells from hypoglycemia and promotes angiogenesis through IGF-1R (insulin-like growth factor type 1 receptor) signaling (126). Conversely, multiple independent studies have clearly demonstrated an angiostatic effect of decorin in the setting of cancer and its down-regulation correlates with the degree of tumor vascularization in various cancer types (99, 100, 108, 127).

The interaction of decorin with VEGFR2 constitutes the most significant contribution to impairing tumor angiogenesis (98, 99) (**Figure 1**). By competing with its canonical¨ ligand VEGFA (vascular endothelial growth factor A), decorin acts as a partial antagonist of VEGFR2 which is specifically located on the surface of endothelial cells to facilitate their angiogenic activity (128–130). Additionally, decorin decreases the proteolytic cleavage of matrix-bound VEGFA in the ECM by inhibiting matrix-metalloprotease activities and expression (99, 107, 131, 132).

Another contributing mechanism of decorin-mediated angiostasis is the recent observation that soluble decorin can reduce VGFA levels by evoking its catabolic degradation *via* autophagy (133) (see below). In parallel to suppressing these pro-angiogenic effectors *via* VEGFR2 signaling, angiogenesis can be modulated by decorin through EGFR. The decorin-EGFR interaction interferes with the signaling cascade that employs Rho (RAS homolog family member A) and the Rho-associated coiled-coil kinase 1 (ROCK1), leading to upregulation of anti-angiogenic effectors, such as thrombospondin-1 (TSP-1) and tissue inhibitor of metalloproteinases-3 (TIMP-3) (108, 131, 132) (**Figure 1**).

Additionally, decorin inhibits angiogenesis by impairing Met signaling, because attenuation of Met signaling causes an induction of TIMP-3. In parallel, hypoxia-inducible factor 1α (HIF-1α) is being repressed and degraded and Met-induced VEGFA expression is inhibited (107, 131). Regulated by a negative feedback loop, loss of HIF-1α in turn inhibits Met expression (131). Collectively, these observations posit the small leucine-rich proteoglycan decorin as an important component of the angiostatic network and the cross-talk between and endothelial and tumor cells.

### 2.3 Decorin Activates Endothelial Autophagy and Cancer Cell Mitophagy

The anti-tumor activities of decorin additionally include the regulation of the autophagy and mitophagy pathways. In vascular endothelial cells, a decorin-induced indirect induction of autophagy was demonstrated by promoting the formation of autophagic initiation complexes and by down-regulation of autophagy inhibitors (134–136). Decorin interaction with VEGFR2 induces the AMP-activated protein kinase (AMPK) and initiates a signaling cascade to activate VPS34 (vacuolar protein sorting 34) and to inhibit mTOR (mammalian target of rapamycin). Decorin evokes sustained autophagy by transcriptional activation of *Peg3* (Paternally expressed gene 3), an imprinted gene expressed exclusively from the paternal allele (137), and that is often silenced in several tumor types by promoter hypomethylation (138, 139). This leads to accumulation of Beclin-1 and LC3 (microtubule-associated protein 1A/1Blight chain 3), which are required for autophagosome formation (130, 136, 140). As Peg3^+^ progenitor cells participate in vascular remodeling (141), and as Peg3 represents a marker for a subset of vessel-associated endothelial progenitors (142), it is possible that decorin may additionally affect angiogenesis *via* Peg3-endothelial stem cells interactions.

A recently uncovered implication of decorin in regulating mitophagy has been reported, which linked decorin directly to affecting the catabolic process of mitophagy within the tumor proper. In line with this, decorin directly activated mitophagy in breast carcinoma cells trough Met receptor activation and subsequent mobilization of PGC-1α (Peroxisome proliferator-activated receptor-γ co-activator 1-α) (143), accompanied by a decorin-mediated mRNA stabilization accumulation of Mitostatin (143) (**Figure 1**). Thus, decorin can affect the cellular energy production and indirectly cell death/apoptosis by evoking autophagic catabolism of mitochondria.

### 2.4 Decorin Regulates Inflammation and the Innate Immune Response

Extensive studies using models for tissue stress and injuries have demonstrated the well-established roles of decorin, and likewise biglycan, in regulating inflammatory and immune responses by interaction with TLR2 and TLR4 (144). There are two mechanisms for decorin-dependent control of inflammation and tumor growth that are based on either stimulation of PDCD4 (programmed cell death protein 4) expression, or on it translational repression (145). PDCD4 is known to promote the inflammatory response by activating NF-κB and suppressing the anti-inflammatory mediator interleukin 10 (IL-10) expression (146). PDCD4 expression is increased by decorin acting as an endogenous ligand of TLR2/4 and stimulating production of proinflammatory molecules, including PDCD4 (145). In a second mechanism, decorin down-regulates the pool of bioactive TGF-β1, which, as mentioned before, can be sequestered by decorin in the ECM. TGF-β1 in turn is an inducer of oncogenic microRNA (miRNA)-21 (147), which functions as a translational repressor of PDCD4 (148). Therefore, decorin indirectly regulates the levels of PDCD4 (145) (**Figure 1**).

## 3 Biglycan Is a Matrix-Derived Signaling Molecule in Cancer

Biglycan, another member of class I SLRPs and structurally homologous to decorin, is a ubiquitously expressed ECM protein (23, 85, 149). The 42 kDa protein core consists of 10 LRRs as well as two N-terminal, covalently bound, tissue-specific CS/DS type GAG chains (23, 150). Through its protein core and GAG chains, biglycan interacts with other ECM proteins like collagen types I-IV and elastin, thereby providing stability and organization in tissues, as well as bone composition (151–156). Although biglycan is tightly bound to the ECM under physiological conditions, the proteoglycan is released from its ECM-bound state during tissue stress and injury through cleavage by proteases (87, 157–160). Additionally, biglycan can be synthesized *de novo* in macrophages during inflammation and is released into circulation (161). In its soluble state, biglycan can be found in the bloodstream in many acute or chronic inflammatory disease acting as a DAMP (93, 159). Biglycan holds great potential as biomarker as deregulated levels of soluble biglycan are detected in a variety of inflammatory and chronic disease, such lupus nephritis (144), diabetes (162, 163), fibrosis (164), renal diseases (159), and cancer (165), biglycan holds great potential as biomarker.

Just as decorin, biglycan has proven to be a versatile matrix-derived signaling molecule that can trigger induction of several pathways involved in tumorigenesis *via* its complex and diverse interactions with both growth factors/cytokines and cell surface receptors (18, 23, 157).

### 3.1 Mechanisms of Biglycan-Induced Inflammation

Under physiological conditions, in its soluble and intact form, biglycan acts as a DAMP and engages in the innate immunity by binding TLR2 and/or TLR4, mimicking gram negative and positive microbial responses and triggering an inflammatory cell response (93, 157). This results in induction of NF-κB and Erk (extracellular signal-regulated kinase) signaling pathways leading to increased levels pro-inflammatory cytokines and chemokines. Most importantly, these include TNF-α and IL-1β, as well as C-C motif ligand (CCL) and C-X-C motif ligand (CXCL) proteins and ultimately result in recruitment of immune cells such as neutrophils, T-cells, B-cells and macrophages into the inflamed regions, infiltrating tissues and organs (23, 161).

**Figure 2:**
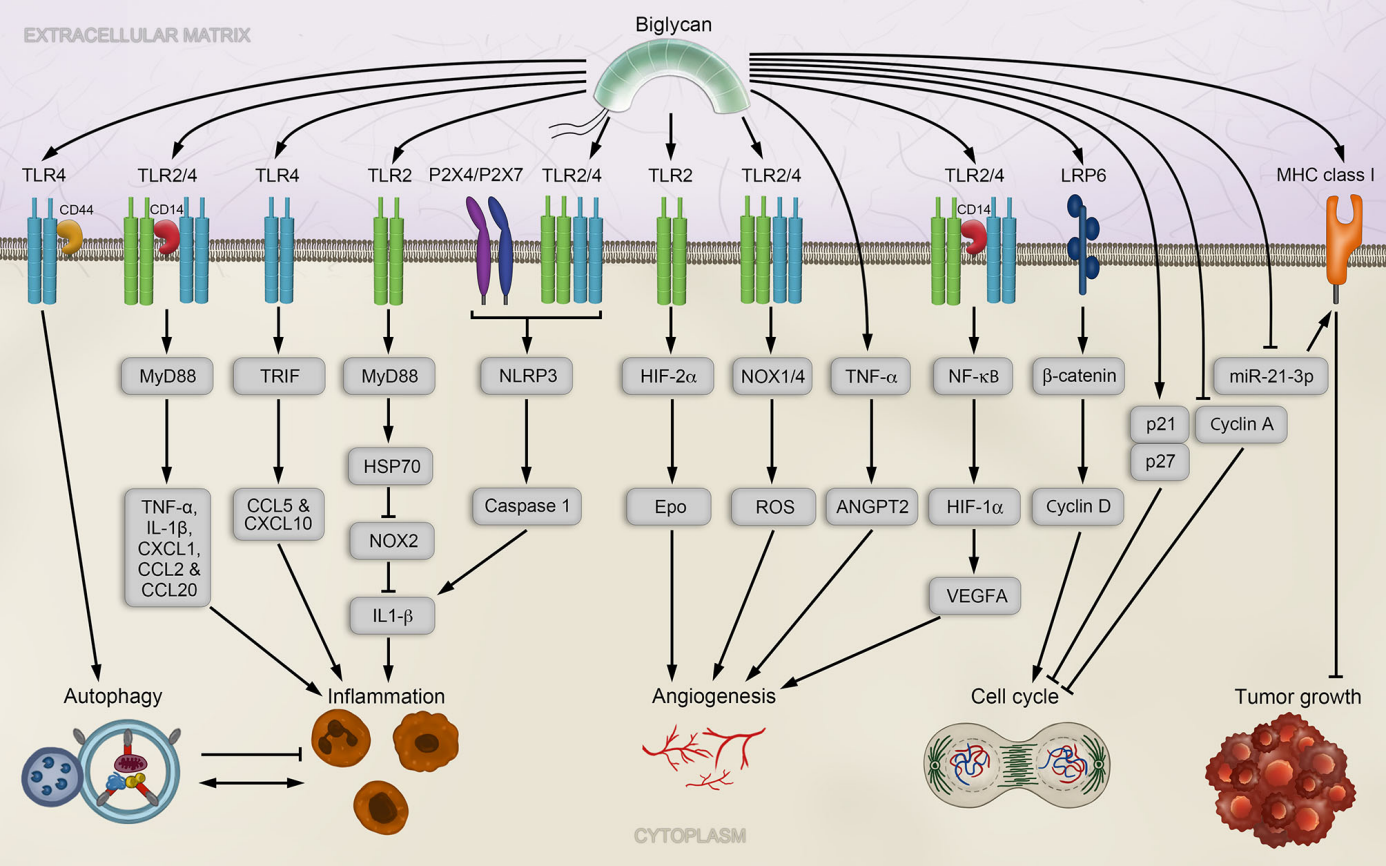
Biglycan signaling and implications in tumorigenesis. Soluble biglycan induces TLR4/CD44mediated autophagy in macrophages and TLR2/4/CD14-mediated inflammation *via* the adaptor molecule MyD88. Inflammation is also trigger by biglycan binding to TLR4 *via* TRIF, or TLR 2 *via* MyD88. Biglycan-mediated clustering of TLR2/4 with the P2X4/P2X7 receptors, which activates the NLRP3 inflammasome, consequently leading to the maturation of IL-1β. In endothelial cells, biglycan promotes angiogenesis in a TLR2/4-dependent manner by enhancing HIF-1α activity and VEGFA expression. Biglycan further affects ROS production *via* TLR2 and TLR4 in a NOX1- and NOX4-dependent manner. Biglycan stabilizes HIF-2α *via* TLR2 interaction and leads to of Epo synthesis. Biglycan knockout impaired tumor by repressing TNF-α and ANGPT2 signaling in a cancer mouse model. Additionally, biglycan can induces a cell cycle arrest by increasing the expression p27 and p21 and decreasing Cyclin A levels. By increasing MHC complex I expression *via* miR-21-3p, biglycan exerts anti-tumoral activities.ANGPT2, angiopoietin 2; CCL, chemokine C–C motif-ligand; CD, cluster of differentiation; CXCL, chemokine C-X-C motif-ligand; Epo, erythropoietin; HIF-1/2α, hypoxia-inducible factor 1/2α; HSP70, heat shock protein 70; IL-1β, interleukin 1 beta; LRP6, low-density lipoprotein receptor-related protein 6; MHC, major histocompatibility complex; miR, microRNA; MyD88, myeloid differentiation primary response 88; NF-κB, nuclear factor kappa-light-chain-enhancer of activated B-cells; NLRP3, NOD-like receptor protein 3; NOX, NADPH oxidase; ROS, reactive oxygen species; TLR2/4, Toll-like receptor 2/4; TNF-α, tumor necrosis factor alpha; TRIF, TIR-domain-containing adapter-inducing interferon-β; VEGFA, vascular endothelial growth factor A.

The biglycan-induced downstream signaling and its outcome depend on the selective utilization of either TLR2 or TLR4, or both, as well as on the interactions with their distinct co-receptors and adaptor molecules (157, 161). For instance, while TLR4/2 interaction with MyD88 specifically induces TNF-α, IL-1β, CXCL1, CCL2, and CCL20, interaction of biglycan with TLR4 and TRIF (TIR-domain-containing adapter-inducing interferon-β) results in expression of CCL5 and CXCL10 (161) (**Figure 2**). Moreover, biglycan interaction with only TLR2 and MyD88 regulates the expression of HSP70 (heat shock protein 70), which binds to NADPH oxidase (NOX)2, and thereby impairs the inhibitory function of NOX2 on IL-1β expression (166) (**Figure 2**). The repertoire of co-receptors employed by TLR2/4 was extended by the discovery of the co-receptor CD14, a high-affinity ligand of biglycan, whose deficiency resulted in abrogated NF-κB, MAPK and ERK signaling and consequent absence of biglycan-induced increase of cytokine expression in macrophages (167).

Additionally, biglycan mediates clustering of TLR2/4 and the purigenic receptors P2X4/P2X7 (168) (**Figure 2**). This enables a cross-talk signaling between both receptor types and allows biglycan to autonomously activate the NLRP3 inflammasome assembly, which is followed by Caspase 1 activation and processing of pro-IL-1β and pro-IL-18 to their mature forms (168).

Biglycan signaling bridges innate and adaptive immune responses as it is involved not only in recruitment of innate immune cells, most importantly macrophages, but also induces synthesis of chemoattractants of B cells and T helper (Th) cells. Mechanistically, the biglycan/TLR4 interaction mediates signaling to sphingosine kinase-1 to regulate the synthesis of macrophage chemoattractants (169). In macrophages and dendritic cells, biglycan/TLR2/4 signaling evokes the expression of a key chemoattractant of B-cells, namely CXCL13 (144). Additionally, biglycan regulates the expression of CXCL9 and CXCL10 *via* TLR4 and TRIF to drive recruitment of Th1 and Th17 cells into the kidney (170). The signaling axis of biglycan and TLR2/4 also enhances antigen-specific T cell activation, potentially in a MyD88/TRIF-dependent manner, and induces autoimmune perimyocarditis (171).

The above-mentioned roles of biglycan in regulating inflammatory signaling and immune response, and in particular the recruitment of immune cells, posit several key connections between biglycan-mediated signaling and cancer-associated inflammation. For instance, the significant role of NLRP3 inflammasome activation in cancer is indisputable but ambivalent and complex (58). Thus, biglycan-mediated NLRP3 inflammasome activation provides an additional regulatory mechanism contributing to its complex regulation. Moreover, CCL2 and CCL5, whose expression is regulated in a biglycan-dependent manner, are known contributors that promote immune cells infiltration at the tumor site, metastasis, and angiogenesis (172–175). In addition, Th1 cells promote synthesis of pro-inflammatory factors, such as IL-12, IL-2, IFNγ and TNFα, thus driving inflammation. Tumor-infiltrating Th1 and Th17 cells have also been identified in various cancers inducing both, pro-tumoral and anti-tumoral immunity (176–178). In line with this, it is possible that biglycan-driven recruitment of immune cells could promote an inflammatory milieu that facilitates tumorigenesis, but also support immuno-surveillance by enhanced Th1, Th17 and macrophage recruitment in some cases with anti-tumorigenic effects of biglycan. These diverse implications of biglycan in triggering and modulating inflammation and the immune response suggest an important role for this proteoglycan in the development of tumor-associated inflammation.

### 3.2 Biglycan as a Regulator of Autophagy

In addition to the various biological roles of biglycan in inflammation, this proteoglycan can cause a switch between inflammation and autophagy in macrophages by selectively binding either CD14 or CD44 with equally high affinities causing an opposing biological outcome. While the interaction of biglycan and CD14, in an TLR2/4-dependent manner, results in an increase of pro-inflammatory signaling and the polarization of M1 macrophages (179), the interaction between biglycan and CD44 counteracts these events and results in the polarization of M2 macrophages as well as in an increase of autophagy-flux and anti-inflammatory effects (180) (**Figure 2**). The unexpected biglycan-induced opposing switch between inflammation and autophagy is solely caused by fluctuations in interaction partners. This process determines whether the downstream signaling will ultimately lead to chronic inflammation, fibrosis and possibly facilitate tumorigenesis, or promote regeneration. These findings underline how tightly those molecular processes, inflammation, and autophagy, are regulated in non-malignant tissues and presumably also in cancer. Accordingly, we presume that a subtle modulation of either one may have major impact on the other, a key factor affecting their therapeutic targeting. The described regulation of immunity responses by biglycan could, for example, affect the resolution of early pre-cancerous lesions, or promote their tumorigenic development. The cause of this switch is yet to be established and future studies will be needed to unravel the precise function of the biglycan-binding epitopes of CD14 and CD44. While these observations were made in non-malignant cells, the fact that biglycan mediates M2 macrophage polarization through autophagy induction suggests that biglycan could be involved in tumor-associated macrophage (TAM) activation during tumor progression at late stages. This is particularly important as sustained biglycan overexpression is linked to enhanced M2 macrophage numbers in ischemia reperfusion injury (180).

Emerging evidence over the past years has demonstrated the crucial role of autophagy in regulating cancer-associated inflammation, and vice versa. For example, autophagy critically affects inflammation by influencing the development, homeostasis, and survival of immune cells, including macrophages, neutrophils and lymphocytes (181, 182). In the future, it will be vital to investigate the mechanisms that regulate the intricate interplay of autophagy and inflammation. Therefore, this recent discovery of CD44 as an additional co-receptor for TLR4 and a high-affinity ligand for biglycan provide novel and valuable insights of possible cross-talk mechanisms between autophagy and inflammation (179, 180).

### 3.3 Mechanisms of Biglycan-Mediated Angiogenesis

Angiogenesis is an important hallmark of cancer and vital for tumor growth and metastasis, as it provides vessels for blood supply and allows transformed cells to enter the circulation (39). Despite their structural homology, the roles of decorin and biglycan in neovascularization are rather opposing. The main mechanism of biglycan signaling employs TLR2 and TLR4 receptors and results in pro-tumorigenic effects, including the production of cytokines and growth factors that ultimately promote tumor angiogenesis. For instance, biglycan-induced, upregulated VEGFA expression promotes neovascularization and cancer growth in colon cancer cells (183). Moreover, biglycan stimulation of human umbilical vein endothelial cells results in increased tubular formation capacity (184). On a molecular basis, biglycan stimulation of endothelial cells results in increased interaction between NF-κB and the HIF-1α promoter in a TLR2/4-dependent manner, enhanced HIF-1α levels and activity, and consequently increased VEGFA expression (184) (**Figure 2**). These findings support a promoting role of biglycan in cancer development and progression, likely by promoting and activating tumor angiogenesis (184).

An additional mechanism that links biglycan signaling to angiogenesis regulation is the enhanced production of reactive oxygen species (ROS). ROS can be produced by NOX enzymes and result in enhanced angiogenesis and tumor growth by increased NF-κB and VEGF expression (185). Due to the ability of biglycan to affect ROS production *via* TLR2 and TLR4 in a NOX1- and NOX4-dependent manner (166, 168) (**Figure 2**), it is very likely that biglycan potentially influences tumor angiogenesis *via* ROS level regulation.

Alternatively, angiogenesis in cancer could be affected by biglycan through the stabilization HIF-2α, which is induced by TLR2 interaction with biglycan and results in the induction of erythropoietin (Epo) synthesis and polycythemia (186) (**Figure 2**). Changes in HIF-2α expression levels have been described in a variety of solid tumors and are linked to TAMs and HIF-2α is, similar to biglycan, associated with late tumor progression stages (187).

In a mouse model of breast cancer with tumor-bearing mice, knockout of biglycan in the stroma inhibited metastasis to the lung, impaired tumor angiogenesis and normalized tumor vasculature by repressing TNF-α and angiopoietin 2 (ANGPT2) signaling (188) (**Figure 2**).

Several studies have focused on the role of biglycan in colon cancer (183, 189, 190). For instance, it was shown that, in colon cancer cells, inhibiting biglycan results in increased expression of pro-apoptotic effectors and is linked to suppressed NF-κB pathway activity. On the other hand, biglycan overexpression does the opposite effect (190). Moreover, biglycan-mediated chemotherapy resistance in colon cancer cells occurs by activating NF-κB signaling (190). Considering the well-established, TLR2/4-dependent function of biglycan in NF-κB signaling, the fact that TLRs have been linked to tumorigenesis of multiple tumor types (191, 192), and the known implications of the NF-κB pathway in the development of chemotherapy resistance in cancers (193, 194), it is convincing that biglycan may promote resistance mechanisms by regulating the NF-κB pathway.

### 3.4 The Role of Biglycan in Promoting Cancer Cell Proliferation, Invasion, and Metastasis

Already a decade ago, a study in osteoprogenitor cells and bone fracture healing models revealed the ability of biglycan to enhance canonical Wnt signaling and to interact with the Wnt co-receptor low-density lipoprotein receptor-related protein 6 (LRP6) (195). Wnt signaling is considered to be a major pathway regulating development and has also been closely linked to carcinogenesis, particularly in the context of colorectal cancer (196, 197). Therefore, these findings suggested a potential function of biglycan in tumorigenesis based on its ability to affect cancer cell proliferation *via* Wnt signaling modulation. More recently, a link between biglycan and Wnt/LRP6 signaling was additionally reported in the context of osteosarcoma. Specifically, biglycan favors MG63 osteosarcoma cell proliferation *via* a LPR6/β-catenin/IGF-1R signaling axis and functions in a positive feedback loop regulating osteosarcoma growth (198). By binding to LRP6, biglycan inhibits the destruction of β-catenin, an inducer of Cyclin D1 expression. Increased Cyclin D1 levels, in turn, activate IGF-1R and ultimately result in enhanced biglycan secretion (198).

Another observation that links biglycan signaling to the regulation of cancer cell proliferation was reported in a study conducted in HCT116 colon cancer cells. In this cellular context, suppression of biglycan expression *via* short hairpin RNA (shRNA) decreases cancer cell proliferation and causes cell cycle arrest at the G0/G1 phase, accompanied by decreased levels of cell cycle associated proteins, including Cyclin A and Cyclin D1 (189). In contrast, expression levels of p21 and p27 are markedly increased in the cancer cells of the control shRNA group (189). Furthermore, the decreased biglycan levels suppressed colon cancer cell migration and invasion, and induced apoptosis in a p38 and MAPK-dependent manner (189).

In the context of gastric cancer, significantly increased tissue biglycan levels have been linked to lymph node metastasis and depth of tumor invasion, both *in vivo* and *in vitro* (199). *In vitro*, investigation of endothelial cell migration, invasion and tube formation ability was positively linked to biglycan levels (199). *In vivo*, biglycan induced focal adhesion kinase (FAK) phosphorylation, suggesting an oncogenic function of biglycan in gastric cancer metastasis in a FAK signaling-dependent manner (199). In line with this, biglycan was recently shown to modulate gastric cancer aggressive features as cell survival, migration, and angiogenesis and biglycan knockout gastric cancer cells showed increased levels of PARP1 (Poly [ADP-ribose] polymerase 1) and Caspase 3 cleavage (200).

Additional roles of biglycan in cancer cell proliferation, migration and metastasis were reported in tumor endothelial cells, where biglycan mediated tumor cell migration *via* TLR2/TLR4/NF-κB/Erk1/2 (201), in melanoma cells, where biglycan increased invasiveness by enhancing integrin-β1 expression (202), and in endometrial cancer cells, where knockdown of biglycan reduced migration, tubular formation and metastasis (203). Collectively, these studies point to a pro-tumorigenic role of biglycan proteoglycan in both mesenchymal (osteosarcoma) and epithelial (colon and breast carcinomas) malignancies.

### 3.5 Anti-Tumorigenic Effects Mediated by Biglycan

The vast majority of research on biglycan in cancer indicate its pro-tumorigenic function. However, several studies have also linked anti-tumorigenic effects to higher biglycan expression levels. Tumor suppressive effects have, for instance, been reported in pancreatic cancer cell lines, where treatment with exogenous biglycan leads to an up-regulation of cyclin-dependent kinase inhibitor p27, down-regulation of Cyclin A and PCNA (proliferating cell nuclear antigen), and cell cycle arrest (204) (**Figure 2**).

Similarly, treatment with exogenous biglycan of bladder cancer cells inhibits cell proliferation and high biglycan levels correlate with prolonged survival of bladder cancer patients (205), and superior patient prognosis of patients with diffuse large B-cell lymphoma (206). In this context, CD40^+^ tumors exhibit increased levels of biglycan that additionally correlate with the number of infiltrating macrophages and CD4^+^ and CD8^+^ T-cells. *In vitro* experiments have demonstrated that CD40 signaling can enhance antigen presentation from malignant B-cells, thus inducing an autologous immune response (207, 208). In line with this, higher biglycan levels could be linked to a high intra-tumoral inflammatory reaction that may result in an increased tumor response, and thereby a better prognosis (206).

In an *in vitro* model of oncogenic transformation, the role of biglycan in the initiation and maintenance of neoplastic transformation was investigated (209). In this study, the HER-2-mediated oncogenic transformation caused silencing of biglycan gene expression, while reconstitution of biglycan expression led to an impaired proliferation and migration of oncogenic transformed cells (209). Therefore, HER2-mediated silencing of biglycan expression may promote tumor cell proliferation and migration and provides a putative therapeutic target for the treatment of HER2^+^ tumor cells (209). Moreover, restoration of biglycan in HER2 fibroblasts decreases their tumorigenic potential when compared to HER2 cells with low biglycan levels (210). Biglycan restoration is linked to an enhanced immune cell responses and increases numbers of immune effector cells in tumors and peripheral blood, possibly resulting from up-regulated major histocompatibility complex (MHC) class I surface antigens, and decreased expression levels of TGF-β and the TGF-β receptor 1 (210). The overexpression of biglycan in HER2 cells additionally mediates an upregulation of decorin, which also elevates MHC class I surface expression in biglycan negative HER2 cells, suggesting a putative synergistic action of both SLRPs (210). Most recently, miR-21-3p mediates down-regulation of MHC class I surface antigens, interferes with the expression of immune-modulating effectors and promotes immune suppression in HER-2/neu cells (211). Induced overexpression of biglycan in HER‐2/neu cells increases MHC class I expression and decreases miR‐21‐3p, highlighting the vital role of biglycan in the MHC class I‐driven immune escape in tumor cells (211) (**Figure 2**).

## 4 Therapeutic Implications and Future Perspectives

Cancer development and progression are multifaceted processes that involve an intricate interplay of genetic and environmental factors. Transformed cells continuously interact with their surroundings, mostly constituted by ECM components. Therefore, proteoglycans, key constituents of the tumor stroma, are increasingly recognized as crucial contributors in shaping the cancer microenvironment by regulating inflammatory and immune responses, affecting autophagy, mediating angiogenesis, and modulating cellular signaling pathways to control cell proliferation, migration and metastasis. All these processes can ultimately either promote resolution and regeneration, or lead to tumor development and disease progression. The diverse implications of proteoglycans in these processes highlight their broad potential as therapeutic targets in cancer therapy. Indeed, the characteristic expression patterns of proteoglycans and their interaction partners in diverse tumor types have been exploited as biomarkers for prognosis and indication for therapy efficiencies.

The well-known pan-tyrosine kinase inhibiting capabilities of decorin have made the SLRP an attractive target for cancer therapies. Although the underlying molecular functions of decorin are complex and not yet fully understood, the evidence favor an antitumorigenic role of decorin. This notion holds great potential of clinical relevance and has led to a variety of studies focused on expressing decorin to attenuate tumorigenic growth (17, 212). Delivery of decorin can be mediated *via* adenoviral vectors or by systemic administration of decorin proteoglycan, protein core or fragments (212). Numerous studies have showed that expression of exogenous decorin can suppress tumor growth, in particular for cancer types that depend on RTK signaling, such as EGFR, Met and IGF-1R (212). Delivery of decorin *via* adenoviral vectors into the tumor cells has been demonstrated to inhibit tumor growth of colon, lung, and squamous cell carcinoma (122, 213). Other decorin gene therapy approaches have been successfully tested in various types of cancer including models of breast cancer (214), glioblastoma (215, 216), prostate tumors (213). These studies have largely confirmed the therapeutic capability of decorin in suppressing tumor development and/or growth (212). Despite the promising evidence from *in vivo* and *in vitro* studies, and although decorin is unlikely to exhibit toxic side effects, the translation into a clinical drug has not been finalized. One major challenge that limits the development of decorin-based clinical drugs are technical limitations in proteoglycan mass production (217). Future improvements in production, delivery and efficiency will improve the rationale for applying decorin in the treatment of cancer.

Over the past few years, our understanding of how biglycan mediated signaling in the state of health and during disease progression has significantly improved. In particular, the recent evidence of the fine-tuned biglycan-mediated regulation of the inflammatory response, which is orchestrated *via* distinct receptor, co-receptor, and adaptor molecules, constitutes an important step towards understanding how biglycan signaling can induce different, and often conflicting, downstream signaling outcomes (218). The biglycan-mediated switch between inflammation and autophagy, depending on the respective co-receptor (167, 180), is of particular interest, but raises several questions that await further investigations. These questions include for example, the identification of the binding stoichiometry of biglycan and its co-receptors CD14 and CD44, of the selection mechanisms that govern the choice of the co-receptors and of the CD14 and CD44 binding motifs. The answers will ultimately enable the translation of the current knowledge of biglycan-mediated signaling into pharmacological interventions, as the ability to precisely modulate the biglycan-induced downstream signaling could be a promising therapeutic approach.

In contrast to decorin, which is associated with mainly anti-tumorigenic effects and where administration of the proteoglycan provides a potent strategy for cancer therapy, biglycan is rather linked to pro-tumorigenic effects. However, TLRs have crucial physiological functions, inhibitory targeting of biglycan-mediated signaling cascades requires further studies to gain precise knowledge on the co-receptors and downstream mechanisms of biglycan/TLR signaling to ensure selective targeting in the context of cancer. In cases where tumor growth is linked to decreased levels of biglycan, delivering biglycan could be beneficial to promote immunogenicity. For example, in the context of HER2 cells, where increased biglycan expression is linked to tumor suppression, the recent discovery of the biglycan/miR-21-3p/MHC class I axis provides an interesting and innovative therapeutic concept for HER2+ cancers by administration of biglycan (211). This could increase immunogenicity mediated by a strong infiltration with effector T cells, macrophages, and support inflammation by the production of pro-inflammatory factors, thus inhibit the escape from immune surveillance. Lastly, it is of note that the regulation of tumorigenesis likely depends on the synergistic and timely action of several components of the ECM and thus it is indispensable to broaden our knowledge on the intricate interplay of these proteoglycans in normal and malignant physiology.

In spite of the rapid progress in this field of research, there are still several outstanding questions. For example, given the cell-dependent context of decorin and biglycan in evoking autophagy in tumorigenesis, at what stage do these proteoglycans play an active role in either suppressing or promoting tumor development? Moreover, do the downstream signaling effects of these autophagic modulators occur independently or are they synergistically coordinated in the tumor stroma? Is decorin vs biglycan more critical in regulating disease-altering autophagy in certain types of malignancies over others? We feel that further investigations into these inquiries are required to better decipher the aberrant matrix remodeling and clarify the disease-driving impact of the ECM in carcinogenesis.

## Author Contributions

All authors listed have made a substantial, direct, and intellectual contribution to the work and approved it for publication.

## Funding

The authors’ laboratories were supported by the German Research Council (SFB 1039, project B02, SFB 1177, 259130777, project E02, all to LS; and the CardioPulmonary Institute (CPI), EXC 2026, Project ID: 390649896 (to LS and MW); WY119/1-3 (to MW), the Else Kroner-Fresenius-Foundation (to MW), and the German Center for Lung Research (to MW);¨ European Union’s Horizon 2020 research and innovation program’s MICROBPREDICT study (No 825694), European Union’s Horizon 2020 Research and Innovation Program GALAXY (No. 668031) and Societal Challenges LIVERHOPE (Health, demographic change, and well-being, No. 731875) the German Research Council (SFB TRR57, CRC1382), Cellex Foundation (PREDICT) all to JT. The original research in Dr. Iozzo laboratory was supported in part by National Institutes of Health Grants RO1 CA39481 and RO1 CA245311.

## Conflict of Interest

The authors declare that the research was conducted in the absence of any commercial or financial relationships that could be construed as a potential conflict of interest.

## Publisher’s Note

All claims expressed in this article are solely those of the authors and do not necessarily represent those of their affiliated organizations, or those of the publisher, the editors and the reviewers. Any product that may be evaluated in this article, or claim that may be made by its manufacturer, is not guaranteed or endorsed by the publisher.

## Glossary

**Table:**

ALR	AIM2-like receptors
AMPK	AMP-activated protein kinase
ANGPT2	angiopoietin 2
CBL	casitas B-lineage lymphoma
CCL	chemokine C–C motif-ligand
CD	cluster of differentiation
CS/DS	chondroitin sulfate/dermatan sulfate
CXCL	chemokine C-X-C motif-ligand
DAMP	damage-associated molecular pattern
ECM	extracellular matrix
EGF	epidermal growth factor
EGFR	epidermal growth factor receptor
EMT	epithelial-mesenchymal transition
Epo	erythropoietin
Erk	extracellular signal-regulated kinase
FAK	Focal-adhesion kinase
GAG	glycosaminoglycan
HGF	hepatocyte growth factor
HIF-1α	hypoxia-inducible factor 1α
HSP70	heat shock protein 70
IGF-1R	insulin-like growth factor type 1 receptor
IL-10	interleukin 10
IL-1β	interleukin 1 beta
LC3	microtubule-associated protein 1A/1B-light chain 3
LRP6	low-density lipoprotein receptor-related protein 6
LRR	leucine-rich repeat
MAPK	mitogen-activated protein kinase
Met	mesenchymal-epithelial transition factor
MHC	major histocompatibility complex
miR	microRNA
mTOR	mammalian target of rapamycin
MYC	myelocytomatosis oncogene protein
MyD88	myeloid differentiation primary response 88
NF-κB	nuclear factor kappa-light-chain-enhancer of activated B-cells
NLRP3	NOD-like receptor protein 3
NLRs	NOD-like receptors
NOX	NADPH oxidase
PARP1	poly [ADP-ribose] polymerase 1
PCNA	proliferating cell nuclear antigen
PDCD4	programmed cell death protein 4
Peg3	paternally expressed 3
PGC-1α	peroxisome proliferator-activated receptor-γ co-activator 1-α
RAS	Ras GTPase/Rat sarcoma
Rho	RAS homolog family member A
ROCK1	Rho-associated coiled-coil kinase 1
ROS	reactive oxygen species
RTK	receptor tyrosine kinase
shRNA	short hairpin RNA
SLRP	small leucine-rich proteoglycan
TGF-β	transforming growth factor β
TGF-β1	transforming growth factor β isoform 1
Th	T helper
TIMP-3	metalloproteinases-3
TLR	Toll-like receptor
TME	tumor microenvironment
TNF-α	tumor necrosis factor alpha
TRIF	TIR-domain-containing adapter-inducing interferon-β
TSP-1	thrombospondin-1
VEGFA	vascular endothelial growth factor A
VEGFR	vascular endothelial growth factor receptor
VEGFR2	vascular endothelial growth factor receptor 2
VPS34	vacuolar protein sorting 34
